# Deep learning classification of lung cancer histology using CT images

**DOI:** 10.1038/s41598-021-84630-x

**Published:** 2021-03-09

**Authors:** Tafadzwa L. Chaunzwa, Ahmed Hosny, Yiwen Xu, Andrea Shafer, Nancy Diao, Michael Lanuti, David C. Christiani, Raymond H. Mak, Hugo J. W. L. Aerts

**Affiliations:** 1 Artificial Intelligence in Medicine (AIM) Program, Mass General Brigham, Harvard Medical School, Boston, MA USA; 2 Department of Radiation Oncology, Dana Farber Cancer Institute and Brigham and Women’s Hospital, Boston, MA USA; 3 Howard Hughes Medical Institute, Chevy Chase, MD USA; 4 Harvard T.H. Chan School of Public Health, Boston, MA USA; 5 Division of Thoracic Surgery, Massachusetts General Hospital, Boston, MA USA; 6 Department of Medicine, Massachusetts General Hospital, Boston, MA USA; 7 Department of Radiology, Dana Farber Cancer Institute and Brigham and Women’s Hospital, Boston, MA USA; 8 Radiology and Nuclear Medicine, CARIM & GROW, Maastricht University, Maastricht, The Netherlands

**Keywords:** Oncology, Cancer imaging, Lung cancer, Biomarkers

## Abstract

Tumor histology is an important predictor of therapeutic response and outcomes in lung cancer. Tissue sampling for pathologist review is the most reliable method for histology classification, however, recent advances in deep learning for medical image analysis allude to the utility of radiologic data in further describing disease characteristics and for risk stratification. In this study, we propose a radiomics approach to predicting non-small cell lung cancer (NSCLC) tumor histology from non-invasive standard-of-care computed tomography (CT) data. We trained and validated convolutional neural networks (CNNs) on a dataset comprising 311 early-stage NSCLC patients receiving surgical treatment at Massachusetts General Hospital (MGH), with a focus on the two most common histological types: adenocarcinoma (ADC) and Squamous Cell Carcinoma (SCC). The CNNs were able to predict tumor histology with an AUC of 0.71(p = 0.018). We also found that using machine learning classifiers such as k-nearest neighbors (kNN) and support vector machine (SVM) on CNN-derived quantitative radiomics features yielded comparable discriminative performance, with AUC of up to 0.71 (p = 0.017). Our best performing CNN functioned as a robust probabilistic classifier in heterogeneous test sets, with qualitatively interpretable visual explanations to its predictions. Deep learning based radiomics can identify histological phenotypes in lung cancer. It has the potential to augment existing approaches and serve as a corrective aid for diagnosticians.

## Introduction

Lung cancer is the leading cause of cancer-related death^1^. It is a heterogeneous disease with many clinically important subtypes^2^. Among these, histologic phenotype is a particularly important predictor of response to therapy and overall clinical outcome^1,2^. More than 80% of all primary lung cancers are classified as non-small cell lung cancer (NSCLC). The major histological types of NSCLC include adenocarcinoma (ADC), and squamous cell carcinoma (SCC); deriving from small and large airway epithelia respectively^1,2^. In clinical practice, manual tissue assessment using conventional light microscopy is a reliable approach for histological categorization^3^. However, biopsy may fail to capture the complete disease morphological and phenotypic profile due to inter- and intra-tumor heterogeneity^4,5^. Moreover, of every tissue block sent for diagnosis, only 1 or 2 slides are assessed^6^, hindering the pathologist’s ability to understand and capture the entire tumor environment^7^. Molecular testing of lung cancers can help capture distinct oncogenic driver mutation profiles for precision oncology^4,5,8–10^, however, the integration of diagnostic molecular pathology into the traditional pathology workflow remains challenging due to the lack of adequate training and expertise, in addition to prohibitive costs^11,12^.

Given the complexity of lung cancer classification and the limitations of current practices, there is a need for innovative clinical data assessment tools to augment the biopsy and help better describe disease characteristics. The automated interpretation of pathology slides through computer-assisted diagnosis (CADx) has the potential to reduce reader variability and is an area of active research^13^. However, despite the emergence of CADx-friendly ecosystems alongside advances in the digitization of 2-dimensional pathology slides as well as 3-dimensional microscopy imaging^13,14^, existing approaches fail to take full advantage of the vast amounts of other data available in modern clinical practice. Histologic classification using routinely acquired radiologic images could have significant implications for diagnostic and treatment decisions.

Radiomics has emerged as a tool for quantifying solid tumor phenotype through the extraction of quantitative radiographic features^15^. There is a growing body of evidence pointing to the prognostic value of such features^5,16,17^ as well as their utility in stratifying patients^18^. While radiomics has primarily relied on the explicit extraction of hand-crafted imaging features^17,19^, more recent studies have shifted towards deep learning—convolutional neural networks (CNNs) specifically—where representative features are learned automatically from data^20–26^. This has fostered the construction of advanced multi-parametric algorithms for cognitive decision-making in many clinical settings^14^. The combination of such powerful computer vision methods with routine medical imaging promises to improve decision-support for the pathologist and oncologist at low cost^16^. Hua, et al*.* implemented deep learning frameworks for pulmonary nodule classification with greater than 70% specificity and sensitivity^21^. A more recent study achieved greater than 99% sensitivity and specificity in lung nodule screening using CT^27^. Xu, et al*.* used deep learning models with time series radiographs to predict pathological response in NSCLC treated with chemoradiation, achieving AUC of up to 0.74^28^. Deep learning based radiomics has also shown promise in other disease sites. Li, et al*.* reported AUC of 0.92 predicting mutational status in low grade gliomas, an improvement on conventional approaches^23^.

In this study, we leverage recent advances in radiomics and deep learning to develop models for enhancing clinician accuracy and productivity within the setting of early-stage NSCLC. Building on data collected through the comprehensive Boston Lung Cancer Survival (BLCS) cohort, we created deep learning models that can act as non-invasive pathological biomarkers for NSCLC. We also found that the CNN-derived CT-radiomics features represented distinct biologic and diagnostic patterns in this cohort and were associated with underlying tumor microanatomy. This preliminary work demonstrates the potential for deep learning based radiomics to enhance the human-based decision tree for NSCLC histology classification.

## Materials and methods

### Data retrieval and selection

**Figure 1:**
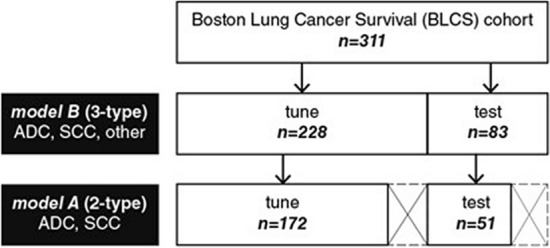
Dataset breakdown for model A and model B. Patients were categorized into three groups; ADC, SCC, and an “Other” category that comprised all other NSCLC histology subtypes. Similar to data presented in Table 2 for model A, model B was fine-tuned using the same BLCS dataset, but with the inclusion of all other histology types. This translated to a tuning-set with 120 ADC, 52 SCC, and 56 patients with “Other” histology types, and a test-set with 35 ADC, 16 SCC, and 32 patients with “Other” histology types (summarized in Fig. 1).

Our model building and validation dataset consisted of a sample of 311 BLCS patients with early-stage NSCLC receiving care at Massachusetts General Hospital (MGH) between 1999 and 2011 (Table 1). Most patients underwent primary surgery for their disease. Approval was obtained from the Mass General Brigham (MGB) Institutional Review Board (IRB# 1999P004935), and written informed consent was obtained on all participants. All methods were carried out in accordance with MGB institutional guidelines and regulations. Pre-resection computed tomography (CT) imaging data was obtained for the patient series. In addition, overall and progression free survival, cancer staging, and histopathologic data corresponding to these patients was documented. All patients had clinical Stage I or Stage II NSCLC. Clinical pathology reports read at MGH were used as ground truth. Patients were categorized into three groups; ADC, SCC and an “Other” category that comprised all other NSCLC histological subtypes, including large cell and mixed histology, bronchoalveolar carcinoma, carcinoid, and cases with more than one primary tumor (Fig. 1). Because oncogenic driver mutation status was not routinely collected for early-stage NSCLC at this site (EGFR/KRAS testing has only been offered since 2008), a small subset of 18 (5.8%) patients had this information available, and no further analysis using molecular data was pursued.

**Table 1:** Patient Characteristics and Follow-up Summary.

Characteristic	Value (n = 311)
Length of Follow-up, Median, yr	3.9
2-year survival, No. (%)	268 (86.2)
Histology, No. (%)	
Adenocarcinoma	155 (49.8)
Squamous Cell Carcinoma	68 (21.9)
Other	88 (28.3)
Stage, No. (%)	
I	186 (59.8)
II	125 (40.2)

^a^ includes all other histology types, specifically, large cell and mixed histology, bronchoalveolar carcinoma, carcinoid, and cases with more than one primary tumor.

Data was partitioned randomly to pick test samples that are representative of the dataset, with no statistically significant difference in characteristics between model fine-tuning and test sets (Table 2). To ensure generalizability, we tested our models on a relatively high proportion of inputs, approximating a 75:25 split.

**Table 2:** Tuning and test dataset characteristics.

Characteristic	Tuning set	Test set	p
**Histology**			
All adeno and SCC^a^ (n = 223)	n = 172 (77%)	n = 51 (23%)	
*Adenocarcinoma*	n = 120 *(70%)*	n = 35 *(69%)*	*p* = *0.892*^**b**^
*Squamous Cell Carcinoma*	n = 52 *(30%)*	n = 16 *(31%)*	*p* = *0.892*^**b**^
**Stage**			
I (n = 129)	n = 102 (59.3%)	n = 27 (52.9%)	*p* = 0.417^**b**^
*A*	n = 61 *(35%)*	n = 13 *(25%)*	
*B*	n = 41 *(23%)*	n = 14 *(27%)*	
II (n = 94)	n = 70 (40.7%)	n = 24 (47.1%)	*p* = 0.586^**b**^
*A*	n = 21 *(12%)*	n = 10 *(20%)*	
*B*	n = 49 *(28%)*	n = 14 *(27%)*	
**Survival**			
2-yr survival	n = 148 *(86%)*	n = 43 *(84%)*	*p* = 0.722^**b**^
**Age**			
Median (yrs)	*67.8*	*68.3*	
**Smoking status**			
Never-smoker	n = 22 (12.8%)	n = 9 (17.6%)	*p* = 0.385^**b**^
Former-smoker	n = 89 *(51.7%)*	n = 27 *(52.9%)*	*p* = 0.881^**b**^
Current-smoker	n = 59 *(34.3%)*	n = 13 *(25.5%)*	*p* = 0.239^**b**^
Not recorded	n = 2 (0.01%)	n = 2 (0.03%)	*p* = 0.917^**b**^
**Sex**^**c**^			
Female/male	n = 97/n = 74	n = 21/n = 30	

Data presented as n, % of respective data set (tuning or test).

^a^Total number of cases with either adenocarcinoma or squamous cell histology, n.

^**b**^p represents the significance of the difference between the two sets.

^**c**^Sex not recorded in one case respectively.

### Image preprocessing

Image pre-processing included manual tumor identification, isotropic rescaling, and density normalization of input CT data. Localization of the tumor regions was performed using clinician-located seed-points. Here, a seed-point is placed in the center of the tumor region using the open-source 3D Slicer software (version 4.5.0–1, https://www.slicer.org/), after assessment of transverse sections slice by slice. We then extract 3D volumes around the seed-points and from this, 2D input tiles measuring 50 mm × 50 mm (Figure S1 in Supplementary Material). Isotropic rescaling was performed on the image data with a linear interpolator to minimize distortion, applying scaling factors that allow for a uniform spatial representation of 1 mm × 1 mm for each isotropic pixel. Density normalization was also performed with mean subtraction and linear transformation.

### Classification with deep convolutional neural-networks

**Figure 2:**
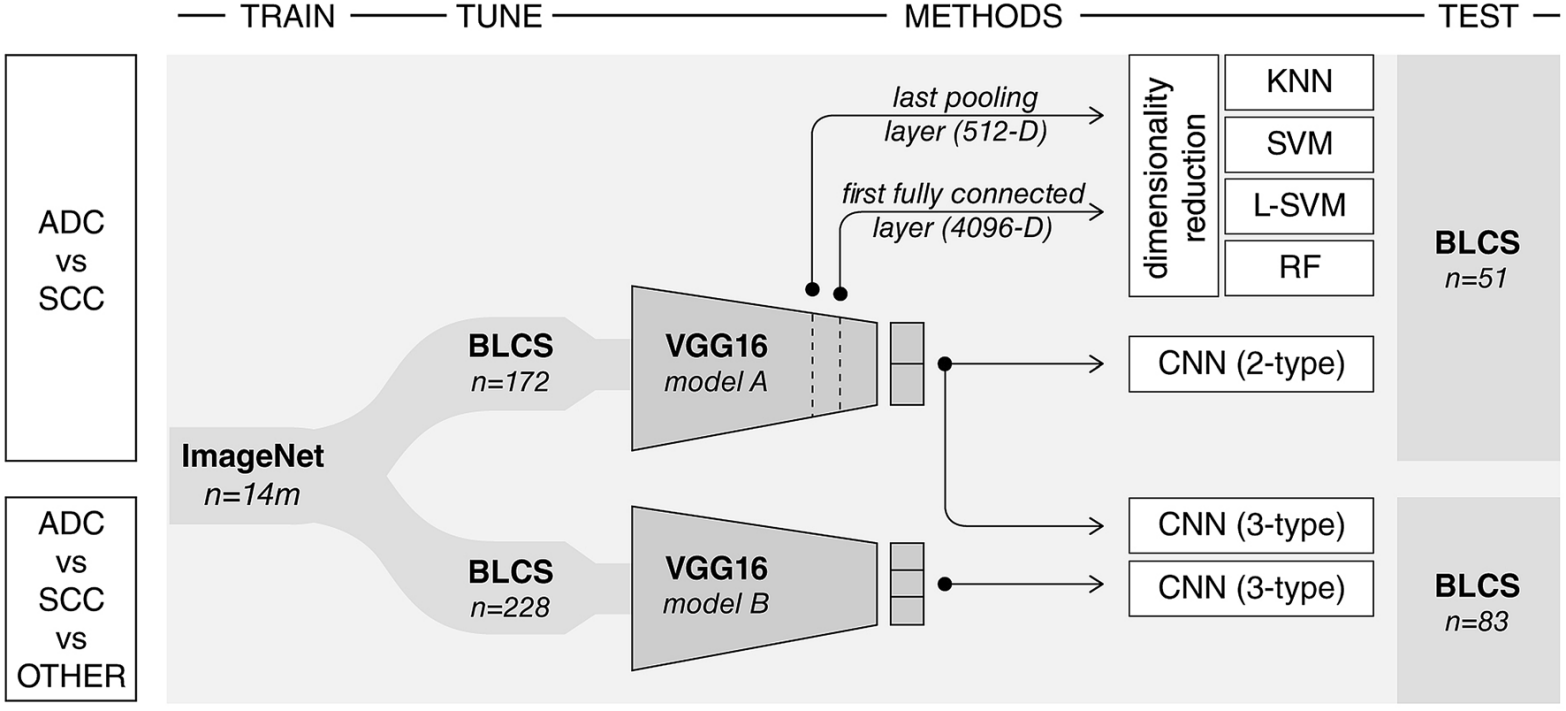
Experimental design. A convolutional neural network (VGG16) developed by the visual geometry group at Oxford (13) and pre-trained on the large ImageNet dataset of more than 14 million hand-annotated natural images is employed in this analytical study. *Model A* is fine-tuned using a sample of 172 patients with either adenocarcinoma or squamous cell carcinoma and is used to predict future cases of these histology types using a held-out test set of 51 patients with adenocarcinoma or squamous cell carcinoma only. This model is also used as a fixed feature extractor for the assessment of machine learning classifiers (kNN, SVM, Linear-SVM, RF). These quantitative radiographic features are derived from the last pooling and first fully connected layers, corresponding to 512-D and 4096-D vectors, respectively. *Model A* is also used as a probabilistic classifier of histology and tested on a held-out test-set of 83 cases containing all histology types, grouped into adenocarcinoma, squamous cell carcinoma, or other. *Model B* is the fully connected VGG16 network tuned with a heterogenous sample of 228 cases with all histology types, and has as its output 3 different histology types, tested on the 83-patient sample as illustrated.

**Figure 3:**
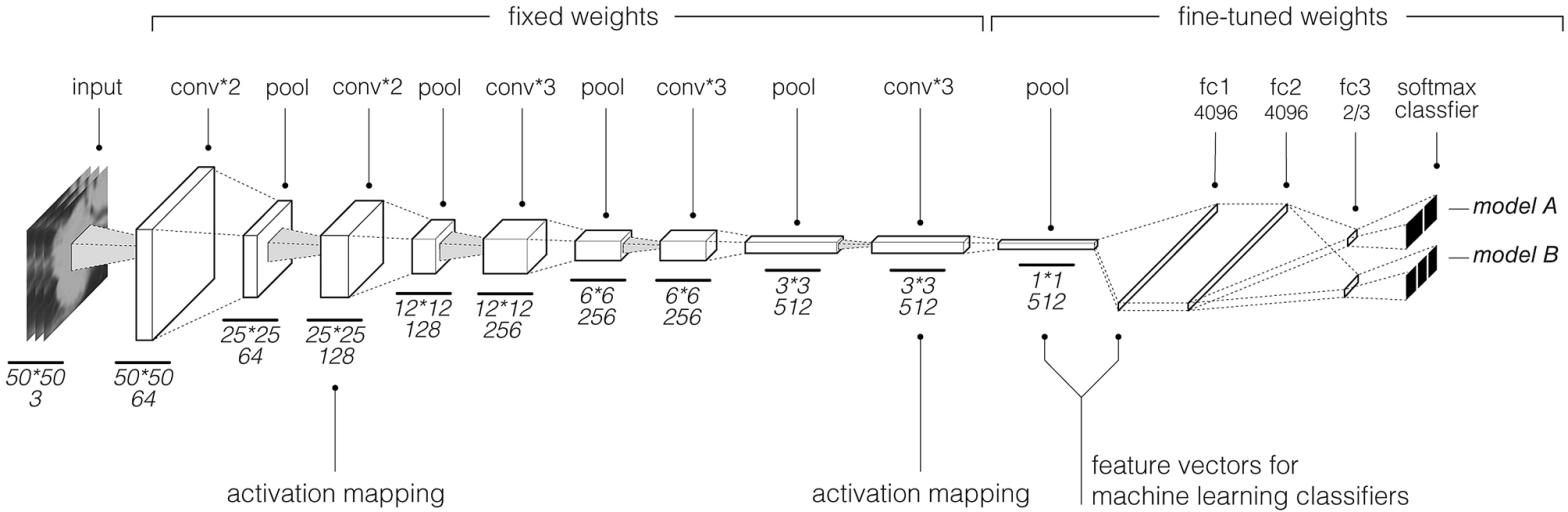
*Model A* and *B* schematic; This convolutional neural network architecture is based on the VGG architecture. With our transfer learning approach, weights of the last convolutional and pooling layers were fine-tuned using radiographic data. Model A, tuned on adenocarcinoma and squamous cell carcinoma tuning-set, had two classes as output in the softmax layer, while Model B which was tuned on a dataset containing all histology types had 3 type outputs.

In this exploratory analysis, CNNs were used for feature extraction and image classification. To address the challenge presented by the scarcity of curated medical data as well as the heterogeneous CT data normally encountered in routine clinical practice, we used a transfer learning approach. Here, robust models that are effective at performing other computer vision tasks are fine-tuned to perform visual recognition on our imaging data. The VGG-16 (Visual Geometry Group) neural network architecture^29^ pre-trained on a large natural image dataset (ImageNet) was assessed. We evaluated the network with fine-tuning of the last convolutional, pooling, and fully connected layers. Hyperparameter optimization was explored iteratively. Inputs of the VGG-16 model were 50 mm x 50 mm image patches. The model had three input channels, all of which were fed grayscale images (that is, model inputs are identical stacked images). Fine-tuning was performed over 100 epochs with a subset of patients that had either ADC or SCC histology for our primary model, *model A*, and with a mix of all 3 histology types (ADC, SCC, and "Other") for the secondary model, *model B* (Fig. 2). Accordingly, the final prediction (softmax) layer was set to 2 for *model A,* and 3 for *model B* (Fig. 3). The predictive performance of the models was evaluated with the area under the receiver operator curve (AUC), and other performance metrics outlined in the model assessment section.

### Feature based analysis and classification

Many studies have shown that CNN-derived feature maps may outperform the original CNN in classification tasks when used with machine learning classifiers such as support vector machine (SVM) and random forest classifiers (RF)^30–32^. Unlike hand-crafted radiomics features, features from CNNs preserve global spatial information with the convolutional kernel operations on the input image^14^. This gives them an advantage in fine-grained recognition, domain adaptation, contextual recognition as well as texture attribute recognition^14^. CNNs are also less dependent on human curation which reduces bias. This provides rationale for an exploratory analysis using the “deep-radiomics” features from our models. For this, we generated features of the tumor regions as represented by the last pooling and the first fully connected layer of *model A*. These abstract high dimensional features are descriptive of the original image data with a great degree of redundancy. The extracted descriptor feature vectors (512-D and 4096-D respectively) were normalized by subtracting the mean, and scaling to unit variance. This is essential to optimize classification performance with discriminative machine learning classifiers, such as SVMs. Despite having flexible criteria, these methods may perform poorly if individual features deviate significantly from a normal distribution. In our data, individual features appeared to follow Gaussian or Gaussian mixture distributions which validates this approach (Figure S2 in Supplementary Material).

Compared to filtered feature reduction techniques which may eliminate important high order features and their relationships, unsupervised feature reduction maintains the interaction among features while eliminating redundant features, benefiting the model training process. Algorithms for unsupervised learning include principal component analysis (PCA) and auto-encoders, a generalized form of PCA. In our analysis, dimensionality reduction was performed using PCA to select independent features corresponding to a set threshold (> 95%) of cumulative explained variance. The least absolute shrinkage and selection operator (LASSO) method was then used to select features that have the strongest association with the target types (shrinkage parameter, α = 0.01). Four machine-learning classification models were independently evaluated on the extracted features: support vector machine (SVM) with both linear and non-linear kernels, k-nearest neighbors (kNN), as well as the random forest (RF) classifier^33,34^.

### Model assessment

We assessed the discriminative power of *model A* in distinguishing the two most common histology types, ADC vs SCC. Tuning for this and the feature-based models was performed on the subset of patients with these histology types, translating to 172 for tuning and 51 for testing. Effects of hyper-parameter optimization e.g. batch size were evaluated, as was the depth of fine-tuning.

To assess the predictive performance of our models we used different descriptive indices including the area under the receiver operator curves (AUC), accuracy, sensitivity, and specificity. We also computed the Wilcoxon rank sum statistic for the binary predicted samples and a two-sided p-value of the test, with the assumption that these are samples from continuous distributions. Features or models with an AUC above 0.60 and a p-value below 0.05 are generally considered predictive in similar studies ^35^.

As a surrogate for how clinically meaningful our imaging-based approach may be, we also performed univariate logistic regression analysis ^36^ for tumor histology using different clinical parameters. Clinical variables that have been observed to have an association with lung cancer and tumor phenotype include age, sex, and smoking status^8,37–43^. Non-binary predictors were standardized by shifting the mean to zero and scaling to unit variance. Smoking status was grouped into never-smokers, current-smokers, and former-smokers (quit at least a year prior). The logistic regression models were built from the same tuning and testing datasets utilized for *model A* (Table 1). AUC and p-value performance metrics in predicting two histology types (ADC vs SCC) were derived in each case for a ready comparison with our deep-learning based model.

A distinct cohort of lung cancer patients treated with surgery (Lung3), which is publicly available at The Cancer Imaging Archive (TCIA) was used as an independent validation dataset ^44,45^. A subset of 49 patients with either ADC or SCC histology was used.

### Neural network prediction probabilities and histological groups

In addition to noting *model A* performance in distinguishing ADC vs SCC, it may also be important to see how our CNN based biomarker performs on a dataset containing other histologies. For this we looked at a heterogeneous held-out test set of 83 patients containing ADC (n = 35), SCC (n = 16), and “Other” histology types (n = 32). Using *model A* as a probabilistic classifier ^46^, the non-parametric Kruskal–Wallis H-test test was performed on the CNN-based prediction probabilities to assess the difference between the three independent samples of ADC, SCC, and “Other” on the test set. A p-value < 0.05 was considered as statistical significance. We also noted the model performance AUC and accuracy for the correct prediction of ADC in this heterogeneous data set (discriminative power).

For comparison, an identical network architecture, *model B* was fine-tuned using a non-overlapping composite dataset of 228 cases with all histology types (ADC, SCC, Other). This separate model was then tested on the same heterogeneous dataset of 83 patients. Given that three types exist for this model, micro-averaging of the predicted types was employed to binarize the ROC scores to either ADC vs all other histologies or SCC vs all other histologies.

### Model interpretability

Activations heat mapping was obtained using Gradient-weighted Class Activation Mapping (Grad-CAM)^47^ with our best performing model, *model A*. Gradient-weighted class activation mapping uses the gradient information flowing into the last convolutional layer of our network to assign importance values to each element in the feature map as it relates to respective class predictions^48^. The rationale behind using the last convolutional layer derives from the fact that deeper layers of a CNN capture higher level visual constructs while retaining spatial information that may be lost in fully connected layers^48^. A combination of the Grad-CAM localizations with the original images provides interpretable visual explanations to model predictions.

## Results

### Clinical characteristics

Our total patient cohort consisted of 311 patients diagnosed with early-stage NSCLC. A total of 186 (59.8%) patients had overall Stage I, and 125 (40.2%) had Stage II disease. Median follow-up from time of diagnosis was 3.5 years, with 86% 2-year survival. 155 (49.8%) patients had pathologist determined ADC, 68 (21.9%) of patients had SCC. The remaining 88 (28.3%) patients had all other histological subtypes, which included large cell and mixed histology, bronchoalveolar carcinoma, carcinoid, and cases with more than one primary tumor. Molecular testing for EGFR/KRAS mutation was done for 18 (5.8%) patients. Overall patient characteristics are summarized in Table 1. *Model A* fine-tuning and test cohort characteristics are summarized in Table 2. For *model B* this translated to a tuning-set with 120 ADC, 52 SCC, and 56 “Other” histology types, and a test-set with 35 ADC, 16 SCC, and 32 “Other” histology types (also summarized in Fig. 1).

### Classification with CNNs

**Figure 4:**
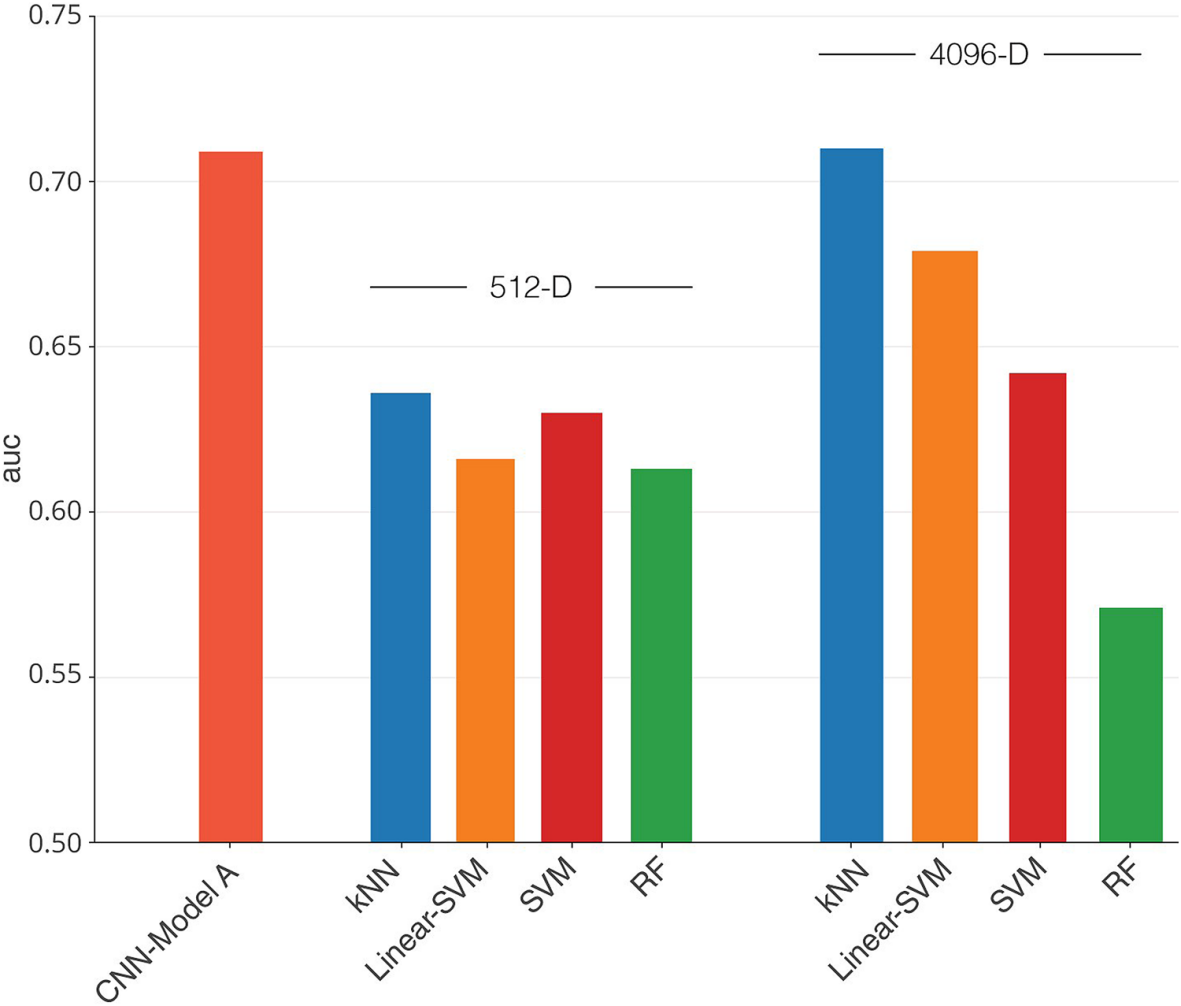
Discriminative performance of deep learning based radiomics models as represented by area under the ROC curve (AUC) scores. Model A, tuned with a dataset containing adenocarcinoma (ADC) and squamous cell carcinoma (SCC) only, displayed an AUC of 0.71 for the 51 patient ADC vs SCC test set, and Model B which was tuned with a dataset containing all histology types had AUC of 0.58 on a heterogenous test set of 83 patients (ADC vs SCC vs Other). Also shown are AUC scores for models combining deep learning derived feature maps with machine learning classifiers. When used on a 4096-D feature vector represented by the first fully connected layer in Model A with dimensionality reduction, the kNN model had an AUC of 0.71, Linear SVM model had AUC of 0.68, SVM model had AUC of 0.64, and RF had AUC of 0.57. When used on a 512-D feature vector, the kNN model had AUC of 0.64, Linear SVM model had AUC of 0.62, SVM model had AUC of 0.63, and RF had AUC of 0.61.

The VGG-16 based *model A* achieved significant predictive performance differentiating between ADC and SCC on a held-out test set of 51 patients with AUC of 0.71 (p = 0.018) (Table 3, Fig. 4, Figure S3A blue in Supplementary Material). Similar fine-tuning and model evaluation was performed with another widely adopted ImageNet architecture, the ResNet50 network architecture^49^. There was no significant difference in its discriminative output and results from this analysis are included in the supplement.

**Table 3:** Histology prediction probabilities for neural network classifier vs CNN-derived feature-based classifiers.

Method	AUC ^a^	Accuracy	Specificity	Sensitivity	p
**Fully connected neural network classifier**					
VGG-16 (Model A)	0.709	68.6%	82.9%	37.5%	0.018
**Machine learning classifiers on 512-D feature vectors**					
kNN (k = 5) ^b^	0.636	68.6%	77.1%	50%	0.123
Linear support vector machine	0.616	70.6%	85.7	37.5%	0.187
Support vector machine	0.630	72.5%	88.6%	37.5%	0.138
Random forest	0.613	72.5%	91.4%	31.3%	0.197
**Machine learning classifiers on 4096-D feature vectors**					
kNN (k = 5) ^b^	0.71	76.5%	85.7%	56.3%	0.017
Linear support vector machine	0.679	74.5%	85.7%	50%	0.042
Support vector machine	0.642	76.5%	97.1%	31.3%	0.107
Random forest	0.571	66.7%	82.9%	31.3%	0.423

^a^Area under the ROC curve. ADC histology corresponds to the “positive” class.

^b^k number of specified nearest neighbors, an even integer.

As a comparison, univariate logistic regression models using clinical parameters yielded AUC of 0.64 (p = 0.118) with smoking status, AUC of 0.55 (p = 0.544) with age, and sex was the strongest predictor of histology in our cohort, with an AUC of 0.69 (p = 0.039). Of note, these findings are consistent with what has been described in the literature, with female and non-smoker predominance in lung adenocarcinoma of young patients^8,37,38^.

*Model A* also demonstrated predictive value with the independent validation dataset (Lung3), achieving AUC of 0.60 (p = 0.251). This dataset contained a sample of 49 patients, of which 30 (61%) had SCC and 19 (39%) had ADC, which is a different skew from the BLCS fine-tuning and test sets. The median age and survival for the Lung3 group was 67.9 years and 3.34 years, respectively.

### Classification with CNN-derived features

With a threshold of 95% cumulative explained variance, PCA was able to perform dimensionality reduction of the 512-D and 4096-D feature space to 60 principal components. Feature selection with the LASSO (alpha = 0.01) yielded the 18 best performing features used in model building.

All models based on CNN-derived features were able to perform binary classification of tumor histology (ADC vs SCC). The 4096-D feature vector seemed to correlate with marginally better predictive performance with most machine learning classifiers. The kNN model had the highest performance (AUC = 0.71, p = 0.017). This was on par with or better than the CNN (AUC = 0.71, p = 0.018). Other classifiers also showed significant predictive power, with an AUC of 0.68 (p = 0.042) for SVC with linear kernel (c = 0.1), AUC of 0.64 (p = 0.107) for non-linear SVC classifier. RF had the lowest predictive performance in all instances (AUC = 0.57, p = 0.423), although this improved to an AUC of 0.61 (p = 0.197) with the 512-D feature vector. All models had higher specificity than sensitivity, while accuracy was again highest with the kNN model (Table 3, Fig. 4).

### Neural network prediction probabilities and histological groups

**Figure 5:**
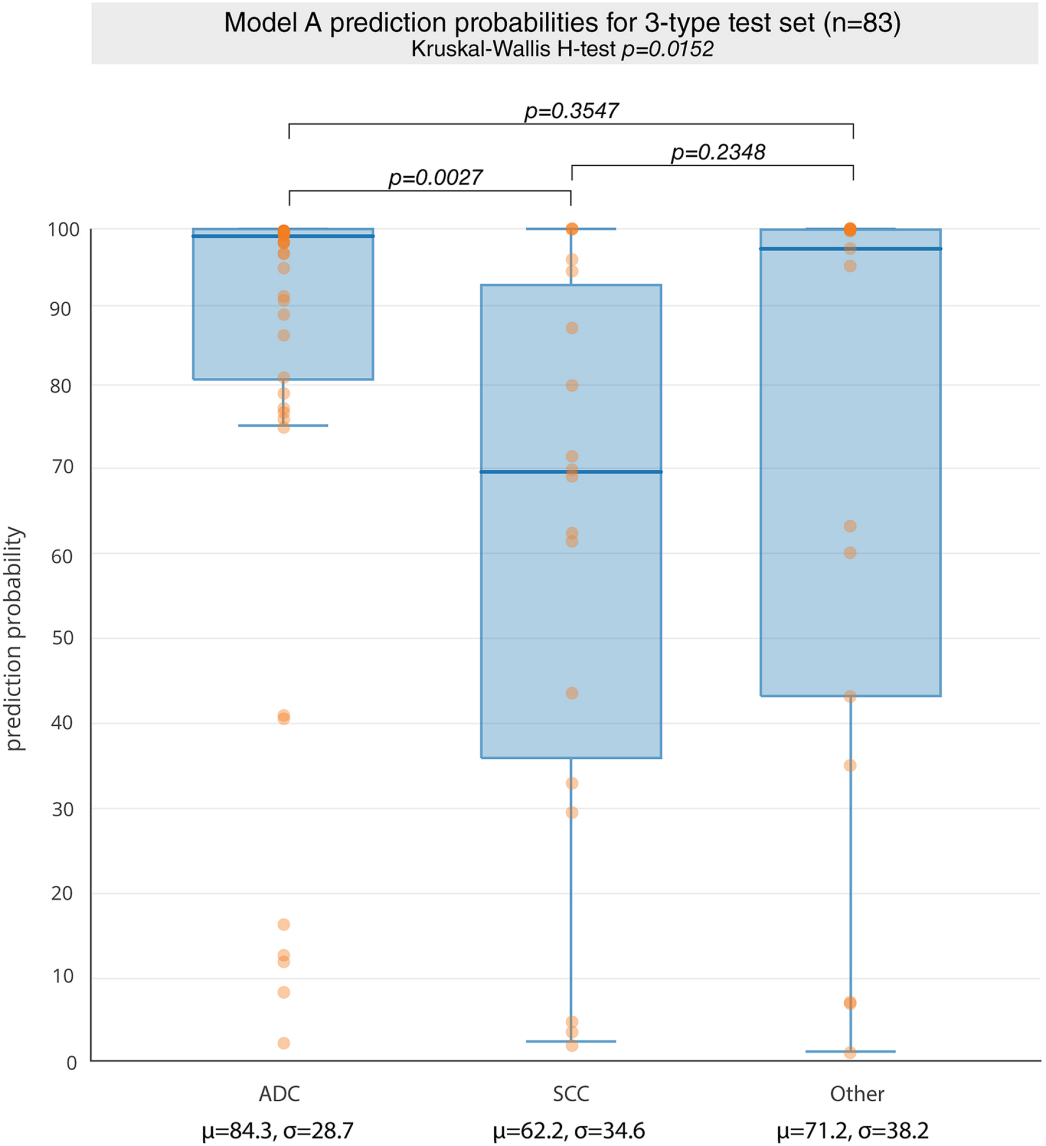
*Model A* as probabilistic classifier of non-small cell histology in 83 sample held-out test set containing all histology types. There is a statistically significant difference in predictions comparing all 3 histology groups: ADC, SCC, Other. Comparison of ADC vs SCC revealed a statistically significant difference with p-value of 0.003, while comparison of SCC vs Other had a p-value of p = 0.235, and ADC vs Other had a p-value of 0.355.

The 83-patient heterogeneous test set contained three histologic subgroups, ADC, SCC, and “Other”. Looking at distributions of the prediction probabilities for each of these subgroups, based on our CNN biomarker, statistically significant difference was noted for a comparison of all 3 groups (p = 0.015). Post-hoc comparisons between groups showed that the difference was most pronounced between the ADC and SCC groups (p-value = 0.003) (Fig. 5). There was a trend towards significance (p = 0.235) between the predictions for the SCC and “Other” groups, however there was no statistically significant difference between the ADC and “Other” groups (p = 0.355). In keeping with the assumption that the test statistic H has a chi-square distribution, our sample sizes were all significantly greater than 5. Even in this heterogeneous test set, *model A* was still able to correctly predict ADC with an AUC of 0.66 (p = 0.013). The test specificity was 85% and sensitivity was 31% for ADC.

A separate analysis using an identical VGG network architecture, *model B* fine-tuned with a heterogeneous tuning set (n = 228) containing all 3 histologic groups also had some predictive power when tested on the same 83 patient test set, albeit to a lesser extent. Using the ROC metric to evaluate classifier output quality for the 3-type model, ROC score when binarizing for SCC vs all other histologies was 0.62 (p = 0.127), and AUC = 0.58 (p = 0.234) when binarizing ADC vs all other histologies (Fig. 4, Figure S3A orange in Supplementary Material). As such, the model trained on ADC and SCC alone outperformed one trained on all histologies in differentiating ADC histology from all other histology types (AUC = 0.66 compared to AUC = 0.58).

### Model interpretability

**Figure 6:**
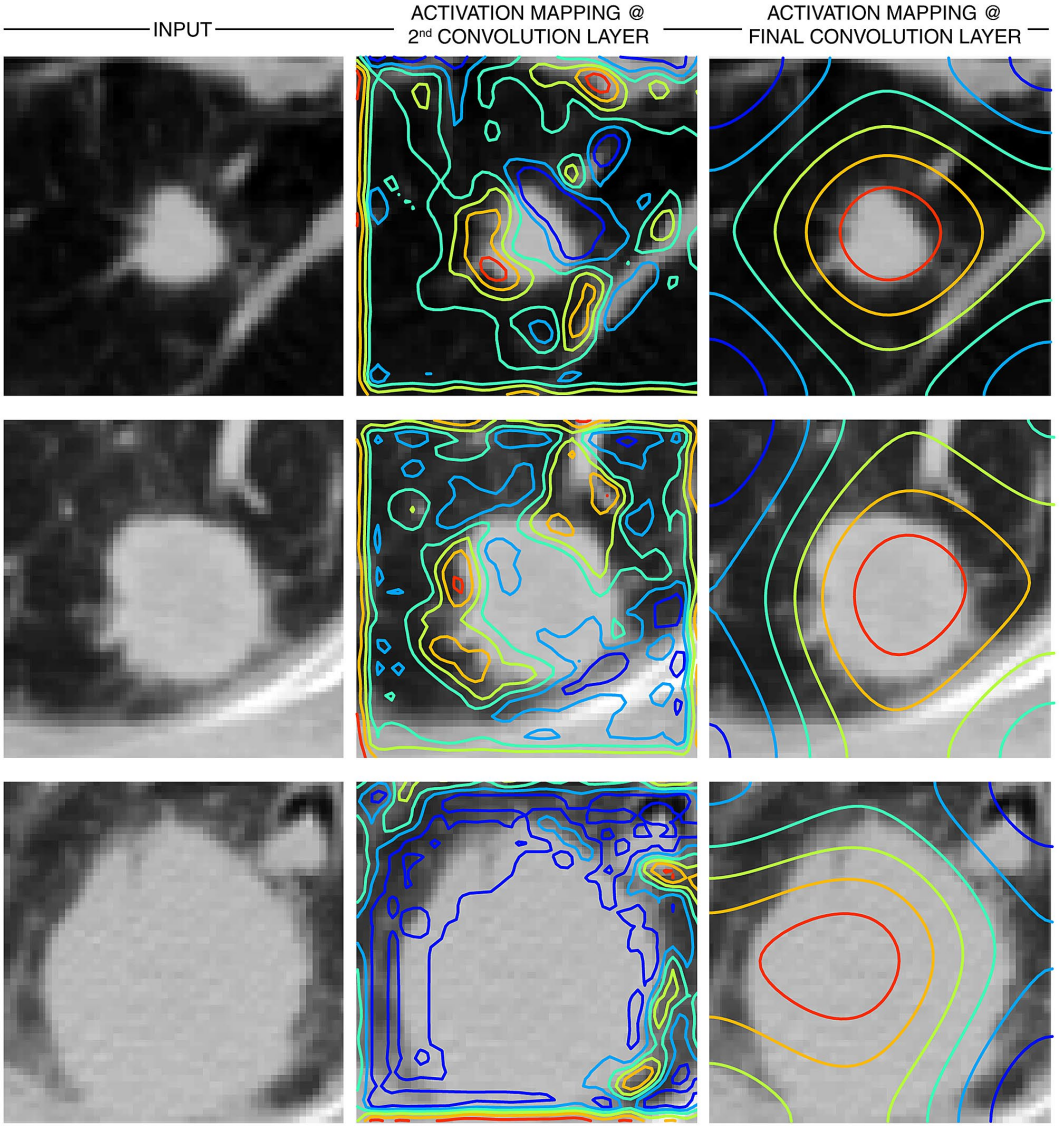
Gradient based class activation heat maps (Grad-CAM) for deep learning based *model A*. Visualization of image regions with the most discriminative value in type prediction as determined by the best performing convolutional neural network model. Here sample test input images are shown with overlaid activation contours, where red highlights regions with highest contribution and blue representing areas with the least value. The second and last convolutional layer in *model A* were used for generation of class activation maps as depicted by Fig. 3.

We extracted Grad-CAM heatmaps for all layers of *model A,* and selected representative examples (Fig. 6). This provided a spatial representation of areas within the input images that contribute the most to the model prediction. The first convolutional layers highlighted tumor edges. This is in line with what is observed when pre-trained models with similar architectures are applied to natural images, while deeper layers tend to pick up more abstract features, and in our experiment highlighted regions on or immediately around the tumor.

## Discussion

We investigated the utility of CNNs in predicting histology in early-stage NSCLC patients, using routinely acquired noninvasive radiologic images. We also assessed the association of CNN-derived quantitative radiographic image feature maps with histologic phenotype in this cohort. The goal of this work was to non-invasively predict lung cancer histology and develop robust deep-learning based radiomics models to help differentiate clinically important histologic subtypes in NSCLC.

We found that CNNs which are effective at natural image recognition tasks, can be implemented to distinguish between the most common histopathologic subtypes in NSCLC. With enough labeled examples, CNNs can detect subtle differences in images to predict phenotypes in future cases^14^. Using pre-trained models enabled us to build on previously learned low-/mid-level features in digital images (e.g., edges, shadows, texture etc.). This reduced the likelihood of over-fitting, given the relatively large models, high dimensionality of features, and the limited size datasets. It also allowed the models to decode heterogeneous image data more effectively, enabling a robustness to variations in routinely acquired clinical data.

Our best performing model was able to detect adenocarcinoma with higher specificity than sensitivity, suggesting greater potential in computer assisted diagnosis, and limited value as a screening tool. Furthermore, there was deterministic signal using this model to predict histology on an independent and different data set, again demonstrating the robustness of the model. The ability to non-invasively predict tumor histology has the potential to boost pathologist accuracy and productivity^14,16^, providing significant cost and time saving benefits.

Prior studies have demonstrated the utility of CNNs as fixed feature extractors for image analysis and classification tasks, with many using the outputs from the last convolutional, pooling, or fully connected layers in VGG or related models^30–32,50^. We followed a similar approach in this work using the image feature representations from these layers in combination with various machine learning classifiers. Narrowing the dimensionality of the deep-radiomics feature space brings performance benefits and avoids over-fitting ^51,52^. This was realized in this study with the kNN estimator which performed on par with the original neural network on the learned features, while other classifiers including SVM also showed significant predictive power with both feature sets. The findings suggest that dimensionality-reduction of CNN derived feature maps to summarize them with low-dimensional vectors, may serve as an effective multi-step alternative to fully-connected neural networks. This approach is in line with similar methods in the data science literature^30–32,53,54^.

Both the 512-D and 4096-D feature vectors were successfully reduced to 18 best performing features. This suggests the same features were selected from both layers, which speaks to the reproducibility of the features. However, machine learning classifiers built around the 4096-D feature vector from the first fully-connected layer seemed to correlate with marginally better predictive performance than from the 512-D feature vector. Neurons in a fully connected layer have full connections to all activations in the previous layer, whereas convolutional layers have connection to only the local features. This could help explain the marginally better performance with the fully connected layer (FC1, Fig. 3).

Looking at our CNN based biomarker as a probabilistic classifier of histology, we found that there is strong association between model prediction value and the likelihood of certain tumor phenotypes being present. That is, higher prediction certainty was associated with correct histology type prediction. For our analysis, because the histology group distribution was unbalanced, with more ADC than SCC and “Other”, we favored using a group-based analysis of prediction probability distributions instead of directly assessing the association of certain types with percentiles of prediction probabilities. The ADC and SCC groups were found to have the most significant difference. This was expected, given our CNN biomarker was trained on distinguishing these two subtypes. No statistically significant difference existed between the ADC and “Other” groups, suggesting a significant overlap in radiographic phenotypes in ADCs and the “Other” group. This is in line with the widely reported misclassification of histology subtypes in these broad umbrella groups, such as the notable misclassification of bronchoalveolar carcinoma (BAC) as adenocarcinoma, undifferentiated NSCLC^55^. Recent revised classification replaces the term BAC altogether^56^. As such, the “Other” group may contain a significant number of misclassified ADCs^2^. These findings not only demonstrate the validity of our CNN biomarker, but also suggest avenues for deep learning-enhanced methods to potentially drive paradigm shifts in histology classification. Adding these “Other” histologies to the test set did introduce noise and reduced our model’s discriminative capacity. Including “Other” histologies in the tuning cohort further reduces model performance, with the model trained on ADC and SCC alone outperforming one trained on all histologies in differentiating ADC histology from all others.

A well-recognized limitation of neural networks is their black-box nature. Looking at intermediate layers may help shed light into learned features, and further enhance the performance of our models. CNN interpretability is an area of increased investigation for the potential to not only help us understand how the models work, but also gain new insights into clinical data and to identify and predict failures. Here we found through gradient-based class activation heat mapping that our best performing model was activating on relevant image regions. In addition to the lesion of interest, our model also highlighted areas around the tumor, suggesting surrounding contextual information may have predictive value. These “at-risk” areas likely correspond to anatomic regions harboring occult microscopic disease that contributes to local treatment failure with therapies such as surgery and radiation. For lesions near the chest wall, the CNN appeared to still focus on the lesion and lung parenchyma, while placing less value on other structures including bone and soft tissue, which may otherwise have similar CT density to tumor. This suggests an ability to learn complex and representative features. Overall, these findings make intuitive sense, and importantly, provide reassurance that the model is detecting the right structures within our region of interest (ROI).

Access to the comprehensive BLCS cohort which has extensive clinical and biologic data was a unique strength of this study. Furthermore, our approach does not rely on accurate volumetric tumor annotations to work. This creates a less time intensive and more efficient workflow, whereas conventional radiomics approaches require precise tumor segmentation, and are therefore more prone to human bias^57,58^. External validation was attained with the independent, “Lung3” surgical cohort. However, some limitations of the present study include small sample size. In addition, the interpretability exercise presented here is qualitative, and quantitative metrics may better validate future analyses, as would experimental design methods that mitigate bias and noise, such as blinding and blocking.

The findings from this exploratory study provide a proof-of-concept that deep-learning based radiomics can identify histological phenotypes in lung cancer, and outperforms clinical parameters such as smoking status, age, and sex at this task. Similar studies have explored using CT texture analysis for histopathological grading in other disease sites including pancreatic ductal adenocarcinoma^59^. While such methods are unlikely to replace the biopsy, there is potential for application as a decision-support tool or corrective aid for the pathologist. Follow up projects will seek prospective validation of our methods using additional large external data sets.

Deep-learning based radiomics has the potential to transform the current rigid classification system into a more analytical and flexible model that includes radiological, biological, and clinical variables^15,17,19,59–62^. There is promise for these methods to augment other emerging techniques, such as liquid biopsy; offering complementary information to guide clinical decision making^62^. However, despite significant advances, challenges for effective integration of these novel tools to routine practice remain. Perhaps most important is the unmet need for wide-ranging data sharing to build large, curated data sets that can be utilized to construct robust and scalable models^63^. Future efforts may benefit from streamlined data mining approaches and the elimination of inter- and intra-institutional data silos. Alternative solutions include federated or collaborative learning, which may enable model training on decentralized data^64^. Such distributed machine learning solutions may help establish stronger correlations between the deep learning based radiomics signatures and tumor biological data.

## Supplementary information

Supplementary information.

## References

[1] Huang T, Li J, Zhang C, Hong Q, Jiang D, Ye M, Duan S (2016). Distinguishing lung adenocarcinoma from lung squamous cell carcinoma by two hypomethylated and three hypermethylated genes: a meta-analysis. PLoS ONE.

[2] Davidson MR, Gazdar AF, Clarke BE (2013). The pivotal role of pathology in the management of lung cancer. J. Thorac. Dis..

[3] Kasraeian S, Allison DC, Ahlmann ER, Fedenko AN, Menendez LR (2010). A comparison of fine-needle aspiration, core biopsy, and surgical biopsy in the diagnosis of extremity soft tissue masses. Clin. Orthop. Relat. Res..

[4] Ilié M, Hofman P (2016). Pros: Can tissue biopsy be replaced by liquid biopsy?. Transl. Lung Cancer Res..

[5] Zhao B, Tan Y, Tsai W-Y, Qi J, Xie C, Lu L, Schwartz LH (2016). Reproducibility of radiomics for deciphering tumor phenotype with imaging. Sci. Rep..

[6] Kohl SK, Lewis SE, Tunnicliffe J, Lott RL, Spencer LT, Carson FL, Souers RJ, Knapp RH, Movahedi-Lankarani S, Haas TS (2011). The College of American pathologists and national society for histotechnology workload study. Arch Pathol Lab Med.

[7] Sun L, Wang D, Zubovits JT, Yaffe MJ, Clarke GM (2009). An improved processing method for breast whole-mount serial sections for three-dimensional histopathology imaging. Am J Clin Pathol.

[8] Aisner DL, Sholl LM, Berry LD, Rossi MR, Chen H, Fujimoto J, Moreira AL, Ramalingam SS, Villaruz LC, Otterson GA (2018). The impact of smoking and TP53 mutations in lung adenocarcinoma patients with targetable mutations-the lung cancer mutation consortium (LCMC2). Clin. Cancer Res..

[9] Rekhtman N, Tafe LJ, Chaft JE, Wang L, Arcila ME, Colanta A, Moreira AL, Zakowski MF, Travis WD, Sima CS (2013). Distinct profile of driver mutations and clinical features in immunomarker-defined subsets of pulmonary large-cell carcinoma. Mod. Pathol..

[10] Schwartzberg L, Kim ES, Liu D, Schrag D (2017). Precision oncology: who, how, what, when, and when not?. Am. Soc. Clin. Oncol. Educ. Book.

[11] Salto-Tellez M, James JA, Hamilton PW (2014). Molecular pathology - the value of an integrative approach. Mol. Oncol..

[12] Fassan M (2018). Molecular diagnostics in pathology: time for a next-generation pathologist?. Arch. Pathol. Lab. Med..

[13] Jansen I, Lucas M, Savci-Heijink CD, Meijer SL, Marquering HA, de Bruin DM, Zondervan PJ (2018). Histopathology: ditch the slides, because digital and 3D are on show. World J. Urol..

[14] Djuric U, Zadeh G, Aldape K, Diamandis P (2017). Precision histology: how deep learning is poised to revitalize histomorphology for personalized cancer care. NPJ. Precis. Oncol..

[15] Gillies RJ, Kinahan PE, Hricak H (2016). Radiomics: images are more than pictures, they are data. Radiology.

[16] Aerts HJWL, Velazquez ER, Leijenaar RTH, Parmar C, Grossmann P, Carvalho S, Bussink J, Monshouwer R, Haibe-Kains B, Rietveld D (2014). Decoding tumour phenotype by noninvasive imaging using a quantitative radiomics approach. Nat. Commun..

[17] Wu W, Parmar C, Grossmann P, Quackenbush J, Lambin P, Bussink J, Mak R, Aerts HJWL (2016). Exploratory Study to identify radiomics classifiers for lung cancer histology. Front. Oncol..

[18] Ganeshan B, Abaleke S, Young RCD, Chatwin CR, Miles KA (2010). Texture analysis of non-small cell lung cancer on unenhanced computed tomography: initial evidence for a relationship with tumour glucose metabolism and stage. Cancer Imag..

[19] Penzias G, Singanamalli A, Elliott R, Gollamudi J, Shih N, Feldman M, Stricker PD, Delprado W, Tiwari S, Böhm M (2018). Identifying the morphologic basis for radiomic features in distinguishing different Gleason grades of prostate cancer on MRI: preliminary findings. PLoS ONE.

[20] Hosny A, Parmar C, Coroller TP, Grossmann P, Zeleznik R, Kumar A, Bussink J, Gillies RJ, Mak RH, Aerts HJWL (2018). Deep learning for lung cancer prognostication: a retrospective multi-cohort radiomics study. PLoS Med.

[21] Hua K-L, Hsu C-H, Hidayati SC, Cheng W-H, Chen Y-J (2015). Computer-aided classification of lung nodules on computed tomography images via deep learning technique. Oncol. Targets Ther..

[22] Hosny A, Aerts HJ, Mak RH (2019). Handcrafted versus deep learning radiomics for prediction of cancer therapy response. Lancet Digit. Health.

[23] Li Z, Wang Y, Yu J, Guo Y, Cao W (2017). Deep Learning based Radiomics (DLR) and its usage in noninvasive IDH1 prediction for low grade glioma. Sci Rep.

[24] Lao J, Chen Y, Li Z-C, Li Q, Zhang J, Liu J, Zhai G (2017). A deep learning-based radiomics model for prediction of survival in glioblastoma multiforme. Sci. Rep..

[25] Rundo F, Spampinato C, Banna GL, Conoci S (2019). Advanced deep learning embedded motion radiomics pipeline for predicting anti-PD-1/PD-L1 immunotherapy response in the treatment of bladder cancer: preliminary results. Electronics.

[26] Afshar, P., Mohammadi, A., Plataniotis, K. N., Oikonomou, A., & Benali, H. From hand-crafted to deep learning-based cancer radiomics: challenges and opportunities. *arXiv [csCV]* (2018) http://arxiv.org/abs/1808.07954

[27] Ali I, Hart GR, Gunabushanam G, Liang Y, Muhammad W, Nartowt B, Kane M, Ma X, Deng J (2018). Lung nodule detection via deep reinforcement learning. Front. Oncol..

[28] Xu Y, Hosny A, Zeleznik R, Parmar C, Coroller T, Franco I, Mak RH, Aerts HJWL (2019). Deep learning predicts lung cancer treatment response from serial medical imaging. Clin. Cancer Res..

[29] Simonyan, K., & Zisserman, A. Very deep convolutional networks for large-scale image recognition. *arXiv [csCV]* (2014). http://arxiv.org/abs/1409.1556

[30] Li Z, Wang Y, Yu J, Guo Y, Cao W (2017). Deep Learning based Radiomics (DLR) and its usage in noninvasive IDH1 prediction for low grade glioma. Sci. Rep..

[31] Notley, S., & Magdon-Ismail, M. Examining the use of neural networks for feature extraction: a comparative analysis using deep learning, support vector machines, and k-nearest neighbor classifiers. *arXiv [csLG]* (2018). http://arxiv.org/abs/1805.02294

[32] Setiono R, Liu H, Liu H, Motoda H (1998). Feature extraction via Neural networks. Feature extraction, construction and selection: a data mining perspective.

[33] Hall P, Park BU, Samworth RJ (2008). Choice of neighbor order in nearest-neighbor classification. Ann. Stat..

[34] Altman NS (1992). An Introduction to Kernel and Nearest-Neighbor Nonparametric Regression. Am. Stat..

[35] Coroller TP, Agrawal V, Narayan V, Hou Y, Grossmann P, Lee SW, Mak RH, Aerts HJWL (2016). Radiomic phenotype features predict pathological response in non-small cell lung cancer. Radiother. Oncol..

[36] Harrell FE, Lee KL, Pollock BG (1988). Regression models in clinical studies: determining relationships between predictors and response. J. Natl. Cancer Inst..

[37] Kim L, Kim KH, Yoon YH, Ryu JS, Choi SJ, Park IS, Han JY, Kim JM, Chu YC (2012). Clinicopathologic and molecular characteristics of lung adenocarcinoma arising in young patients. J. Kor. Med. Sci..

[38] Saito S, Espinoza-Mercado F, Liu H, Sata N, Cui X, Soukiasian HJ (2017). Current status of research and treatment for non-small cell lung cancer in never-smoking females. Cancer Biol. Ther..

[39] Blandin Knight S, Crosbie PA, Balata H, Chudziak J, Hussell T, Dive C (2017). Progress and prospects of early detection in lung cancer. Open Biol..

[40] Hecht SS (1999). Tobacco smoke carcinogens and lung cancer. J. Natl. Cancer Inst..

[41] Hu Y, Chen G (2015). Pathogenic mechanisms of lung adenocarcinoma in smokers and non-smokers determined by gene expression interrogation. Oncol. Lett..

[42] Brown JS, Eraut D, Trask C, Davison AG (1996). Age and the treatment of lung cancer. Thorax.

[43] Pinsky PF, Berg CD (2012). Applying the National Lung Screening Trial eligibility criteria to the US population: what percent of the population and of incident lung cancers would be covered?. J. Med. Screen.

[44] Clark K, Vendt B, Smith K, Freymann J, Kirby J, Koppel P, Moore S, Phillips S, Maffitt D, Pringle M (2013). The Cancer Imaging Archive (TCIA): maintaining and operating a public information repository. J. Digit. Imaging.

[45] Aerts HJWL, Hugo JW, Velazquez ER, Leijenaar RTH, Parmar C, Grossmann P, Carvalho S, Bussink J, Monshouwer R, Haibe-Kains B (2014). Decoding tumour phenotype by noninvasive imaging using a quantitative radiomics approach. Nat. Commun..

[46] Garg, A., & Roth, D. Understanding Probabilistic Classifiers. In *Machine Learning: ECML 2001* (Springer, Berlin), pp. 179–191.

[47] Selvaraju, R. R., Cogswell, M., Das, A., Vedantam, R., Parikh, D., & Batra, D. Grad-CAM: visual explanations from deep networks via gradient-based localization. in *2017 IEEE International Conference on Computer Vision (ICCV)*, 618–626.

[48] Selvaraju, R. R., Cogswell, M., Das, A., Vedantam, R., Parikh, D., & Batra, D. Grad-CAM: visual explanations from deep networks via gradient-based localization. *arXiv [csCV]* (2016) http://arxiv.org/abs/1610.02391

[49] He, K., Zhang, X., Ren, S., & Sun, J. Deep residual learning for image recognition. in *Proceedings of the IEEE conference on computer vision and pattern recognition* (pp. 770–778).

[50] Zeiler MD, Fergus R (2014). Visualizing and Understanding Convolutional Networks. Computer Vision – ECCV 2014.

[51] Hastie T, Tibshirani R, Friedman J (2009). The Elements of Statistical Learning: Data Mining, Inference, and Prediction.

[52] James G, Witten D, Hastie T, Tibshirani R (2013). An Introduction to Statistical Learning: with Applications in R.

[53] Mafarja M, Mirjalili S (2018). Whale optimization approaches for wrapper feature selection. Appl. Soft. Comput..

[54] Islam MMM, Islam MR, Kim J-M (2017). A hybrid feature selection scheme based on local compactness and global separability for improving roller bearing diagnostic performance. Artificial Life and Computational Intelligence.

[55] Raz DJ, Zell JA, Karnezis AN, Odisho A, Ignatius Ou SH, Anton-Culver H, Jablons DM (2006). Misclassification of bronchioloalveolar carcinoma with cytologic diagnosis of lung cancer. J. Thorac. Oncol..

[56] Gardiner N, Jogai S, Wallis A (2014). The revised lung adenocarcinoma classification-an imaging guide. J. Thorac. Dis..

[57] Joskowicz L, Cohen D, Caplan N, Sosna J (2018). Automatic segmentation variability estimation with segmentation priors. Med Image Anal..

[58] Zhao B, Tan Y, Bell DJ, Marley SE, Guo P, Mann H, Scott MLJ, Schwartz LH, Ghiorghiu DC (2013). Exploring intra- and inter-reader variability in uni-dimensional, bi-dimensional, and volumetric measurements of solid tumors on CT scans reconstructed at different slice intervals. Eur. J. Radiol..

[59] Qiu W, Duan N, Chen X, Ren S, Zhang Y, Wang Z, Chen R (2019). Pancreatic ductal adenocarcinoma: machine learning-based quantitative computed tomography texture analysis for prediction of histopathological grade. CMAR.

[60] Austin JHM, Garg K, Aberle D, Yankelevitz D, Kuriyama K, Lee H-J, Brambilla E, Travis WD (2013). Radiologic implications of the 2011 classification of adenocarcinoma of the lung. Radiology.

[61] Coroller TP, Bi WL, Huynh E, Abedalthagafi M, Aizer AA, Greenwald NF, Parmar C, Narayan V, Wu WW, Miranda de Moura S (2017). Radiographic prediction of meningioma grade by semantic and radiomic features. PLoS ONE.

[62] Parekh VS, Jacobs MA (2019). Deep learning and radiomics in precision medicine. Expert. Rev. Precis. Med. Drug Dev..

[63] de Fortuny EJ, Martens D, Provost F (2013). Predictive modeling with big data: is bigger really better?. Big Data.

[64] Bonawitz, K., Eichner, H., Grieskamp, W., Huba, D., Ingerman, A., Ivanov, V., Kiddon, C., Konečný, J., Mazzocchi, S., Brendan McMahan, H., et al. Towards Federated Learning at Scale: System Design. *arXiv [csLG]* (2019) Available at: http://arxiv.org/abs/1902.01046

