# Supplementary material for: Deep learning classification of lung cancer histology using CT images
Source: Sci Rep. 2021 Mar 9;11:5471. doi: 10.1038/s41598-021-84630-x (PMC7943565; doi:10.1038/s41598-021-84630-x)
Supplement: Supplementary file 1 — Supplementary information. [file 41598_2021_84630_MOESM1_ESM.pdf]

Deep learning classification of lung cancer histology using CT images

Tafadzwa L. Chaunzwa, MD[1,2]; Ahmed Hosny, MS[1]; Yiwen Xu, PhD[1]; Andrea Shafer, MPH[3]; Nancy Diao, SD[3]; Michael Lanuti, MD[4]; David C. Christiani, MD[3,5]; Raymond H. Mak, MD[1]; Hugo JWL. Aerts, PhD[1,6]

1. Department of Radiation Oncology, Dana Farber Cancer Institute and Brigham and Women's Hospital, Boston, MA
2. Howard Hughes Medical Institute, Chevy Chase, MD
3. Harvard T.H. Chan School of Public Health, Boston, MA
4. Division of Thoracic Surgery, Massachusetts General Hospital, Boston, MA
5. Department of Medicine, Massachusetts General Hospital, Boston, MA
6. Department of Radiology, Dana Farber Cancer Institute and Brigham and Women's Hospital, Boston, MA

Please address all correspondence to hugo_aerts@dfci.harvard.edu and/or tafadzwa_chaunzwa@dfci.harvard.edu.

FIGURE S1: Seed points based tumor region segmentation.

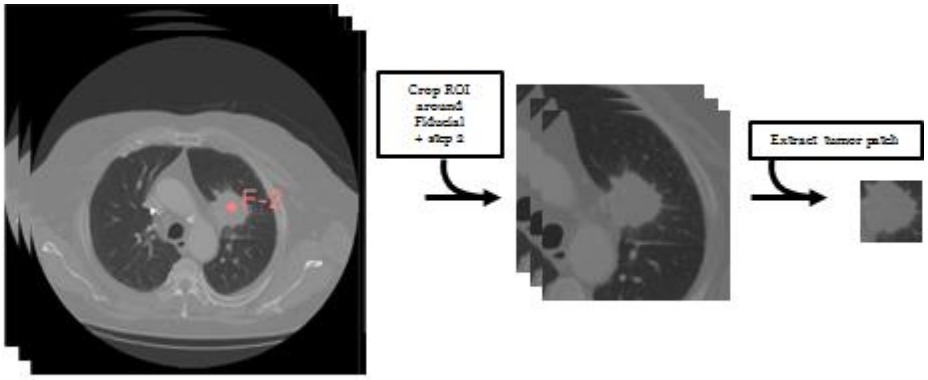

FIGURE S2: Deep-radiomics features in both training and test data follow Gaussian or Gaussian Mixture distributions.

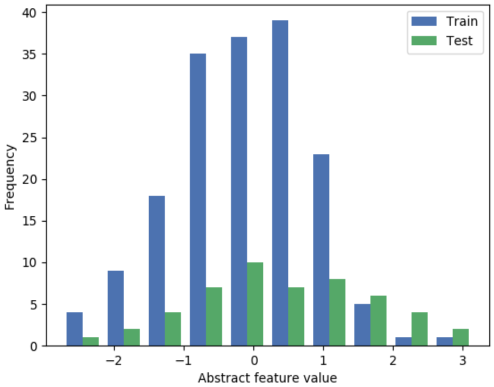

Figure S3: Discriminative performance of deep learning based computed tomography radiomics models. (A) The receiver operating characteristic curves (ROC) plotting deep-learning model performance for differentiating non-small cell lung cancer histology using radiographic data. Model A tuned with a dataset containing adenocarcinoma and squamous cell carcinoma only, displayed an area under the ROC curve (AUC) of 0.71 for the 51 patient test set, and Model B which was tuned with a dataset containing all histology types had AUC of 0.58 on a heterogenous test set of 83 patients. (B) ROC curves for deep learning derived feature maps used in combination with machine learning classifiers. When used on a 4096-D feature vector represented by the first fully connected layer in Model A with dimensionality reduction, the kNN model had an AUC of 0.71, Linear SVM model had AUC of 0.68, SVM model had AUC of 0.64, and RF had AUC of 0.57.

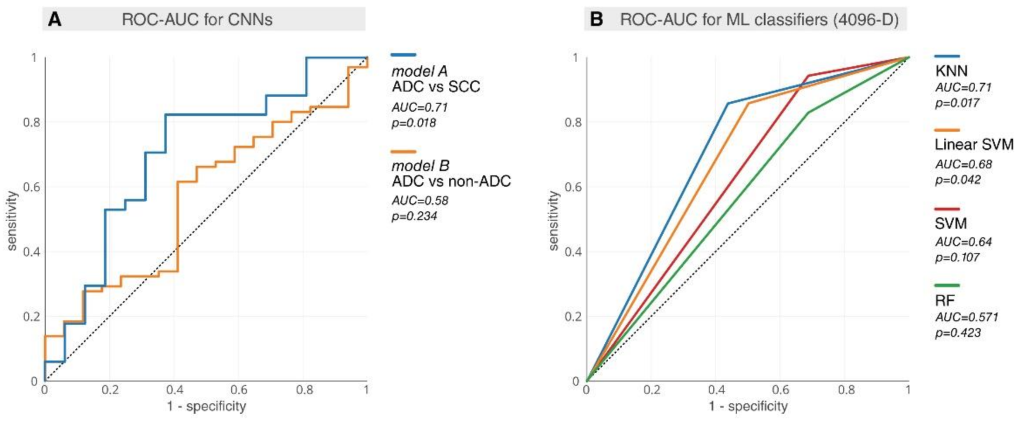

Abbreviations: CNN, convolutional neural network; SCC, squamous cell carcinoma; ADC, adenocarcinoma; SVM, support vector machine; kNN, k-nearest neighbor; RF, random forest; PCA, principal component analysis; ROC, receiver operating-characteristic curve; AUC, area under the ROC curve; NSCLC, non-small cell lung cancer, VGG, visual geometry group network architecture; LASSO, least absolute shrinkage and selection operator; CAD, computer-assisted diagnosis; EGFR, epidermal growth factor receptor; KRAS, Kirsten rat sarcoma viral oncogene; ROI, region of interest

VGG-16 neural network

The VGG architecture, developed by the visual geometry group at Oxford (1), makes an improvement over prior deep-learning models by introducing multiple stacked 3 x 3 kernel sized filters in place of large filters. This increases the depth of the neural

network, enabling it to learn complex features. The architecture has blocks with the same filter size applied multiple times to extract more complex and representative features.

Machine Learning Classifiers

SVM

Support vector machines demonstrate high precision and robustness, and have been used in many cancer studies (14). In our analysis both linear and non-linear kernels for the SVM were evaluated. The box constraint C was set to 0.1 for the linear kernel.

kNN

In this weighted neighborhood scheme, object classification is based off the majority vote among its k nearest neighbors by Euclidean distance in feature space (2,3). The Nearest Neighbor classifier which is associated with multiple hyper-parameters, e.g using pixel wise differences to compare two images. Representing the images as vectors I1 and I2, one can compare them using the L1, and L2 distances:

$$\mathrm{d}_1(I_1, I_2) = \sum_p |I_1^p - I_2{}^p| \ , \quad \mathrm{d}_2(I_1, I_2) = \sqrt{}\sum_p \left(I_1^p - I_2{}^p\right)^2$$

We set k = 5 for our model.

Random Forest Classifier

This ensemble technique can be viewed as an adaptive weighted nearest neighbors, formalized by a weight function, W (4,5):

$$f(x) = \sum_{j=1}^{N} W(x_j, x')y_j$$

LASSO

LASSO performs both variable selection and regularization in order to enhance the prediction accuracy and interpretability of the statistical model it produces (4,5). The lasso estimate is defined by:

$$RSS(D) = D_0 + \sum_{j=1}^{N} \left(y_j - D_0 - \sum_{i=1}^{M} x_{ji} D_j \right)^2$$

Least squares estimation in which we pick coefficient D= (D0, D1,…, DN) to minimize the residual sum of squares:

$$D^{lasso} = D_0 + \sum_{j=1}^{N} \left(y_j - D_0 - \sum_{i=1}^{M} x_{ji} D_j \right)^2 + L \sum_{j=1}^{M} |D_j|$$

Supplemental analysis with residual neural-network (ResNet50)

Residual networks allow for training of very deep convolutional neural networks, bypassing limitations associated with degradation and vanishing gradients (6). This architecture allows for improved accuracy, without increased training errors. Here we trained the ResNet50 model over 100 epochs with fine tuning of the last convolutional layers. The same subset of patients used for the VGG-16 model as outlined in the manuscript was used for training, cross-validation, and testing.

Similar fine tuning was performed for the “very” deep ResNet50 network architecture. Inputs for the ResNet50 model the same images were extrapolated to best fit optimal input dimensions for this network (224x224 pixels) We evaluated the network with fine-tuning of the last convolutional layers, as well as the fully connected layers. The final prediction (softmax) layer was changed from 1000 to 2 to predict the 2 main histology types as with the VGG network in the primary analysis.

The model was able to demonstrate discriminative ability between ADC and SCC with using non-invasive CT data, with an AUC of 0.77. There was only a small performance improvement using the ResNet50 architecture over the VGG-16 model. Given the drawbacks of very deep neural networks, such as the need for more data to avoid overfitting, and the differential performance changes noted here, smaller models may be best suited for this visual recognition task. In addition, the model’s inflexibility with input

image size compared to the VGG network limited its utility, particularly with regards to the small lesions encountered in the present study.

References

1. Simonyan K, Zisserman A. Very Deep Convolutional Networks for Large-Scale Image Recognition. arXiv [csCV] (2014) Available at: http://arxiv.org/abs/1409.1556

2. Altman NS. An Introduction to Kernel and Nearest-Neighbor Nonparametric Regression. The American Statistician 1992:46(3): 175–185. doi:10.1080/00031305.1992.10475879

3. Hall P, Park BU, Samworth RJ. Choice of neighbor order in nearest-neighbor classification. Ann. Statist. 2008: 36(5) ; 2135-2152

4. Hastie T, Tibshirani R, Friedman J. The Elements of Statistical Learning: Data

Mining, Inference, and Prediction 2nd Edition. (2009)

5. James G, Witten D, Hastie T, Tibshirani R. An Introduction to Statistical Learning with Applications in R. (2013)

6. He K, Zhang X, Ren S, Sun J. Deep residual learning for image recognition. in 577 Proceedings of the IEEE conference on computer vision and pattern recognition, 578 770–778
